# Coverage of Vitamin A Capsule Programme in Bangladesh and Risk Factors Associated with Non-receipt of Vitamin A

**DOI:** 10.3329/jhpn.v28i2.4884

**Published:** 2010-04

**Authors:** Richard D. Semba, Saskia de Pee, Kai Sun, Nasima Akhter, Martin W. Bloem, V.K. Raju

**Affiliations:** ^1^ Wilmer Eye Institute, Johns Hopkins University School of Medicine, Baltimore, MD, USA; ^2^ Nutrition and HIV/AIDS Policy, Strategy and Programme Support Division, World Food Programme, Rome, Italy; ^3^ UCL Centre for International Health and Development, London, United Kingdom; ^4^ Eye Foundation of America, Morgantown, WV, USA

**Keywords:** Blindness, Child, Morbidity, Mortality, Risk factors, Vitamin A, Vitamin A deficiency, Bangladesh

## Abstract

Vitamin A supplementation reduces child morbidity, mortality, and blindness. The coverage of the national vitamin A programme and risk factors for not receiving vitamin A were characterized using data from the Bangladesh Demographic and Health Survey 2004. Of 3,745 children aged 18–59 months, 3,237 (86.4%) received a vitamin A capsule each within the last six months. Children who missed vitamin A were more likely to be stunted (prevalence ratio [PR] 0.97, 95% confidence interval [CI] 0.95–1.00) and come from a family with a previous history of mortality of children aged less than five years (PR 0.95, 95% CI 0.91–0.99). Maternal education of ≥10 years (PR 1.09, 95% CI 1.04–1.13), 7–9 years (PR 1.08, 95% CI 1.04–1.12), and 1–6 years (PR 1.05, 95% CI 1.02–1.08) compared to no formal education was associated with the child not receiving vitamin A in a multivariate model, adjusting for potential confounders. Children missed by the vitamin A programme were more likely to come from families with lower maternal education. Special efforts are required to ensure that the coverage of the national vitamin A programme is increased further so that the most vulnerable children are also better protected against morbidity, mortality, and blindness.

## INTRODUCTION

Vitamin A deficiency is a major public-health problem in Bangladesh, where it is a leading cause of morbidity, mortality, and blindness among preschool children (1–3). Vitamin A is found as retinyl esters as in egg-yolk, whole milk, butter, and liver, or as provitamin A carotenoids, as in dark green-leafy vegetables, carrots, and red/orange/yellow-coloured fruits. Vitamin A deficiency can result in more severe infections and greater mortality due to diarrhoea and measles, especially in preschool children because vitamin A is essential for normal immune function. It is also essential for growth, reproduction, and vision (4).

Many developing countries worldwide have established programmes to provide periodic supplementation of high-dose vitamin A to increase child survival and reduce the incidence of nutritional blindness. Vitamin A supplementation is one of the most cost-effective interventions for child health (5) and is known to reduce mortality of preschool children by nearly one-quarter (6). The Millennium Development Goals (MDGs) include reducing child mortality by two-thirds between 1990 and 2015 (5, 7), and the effective coverage of periodic supplementation programmes of high-dose vitamin A is considered one of the most cost-effective strategies in reaching this goal. The specific aims of this study were to characterize the coverage of the national vitamin A capsule programme in Bangladesh and to identify the factors associated with receipt or non-receipt of a vitamin A capsule within the last six months. For these, we examined population-based, demographic and health survey data from Bangladesh.

## MATERIALS AND METHODS

The study subjects included preschool children and their families who participated in the Bangladesh Demographic and Health Survey (BDHS) 2004, a nationally representative survey of 10,500 households selected from 361 clusters throughout Bangladesh (8). The primary objective of the survey was to provide data to monitor the population and health situation in Bangladesh. The survey used a multistage cluster sample that was based upon the 2001 Bangladesh Census, and it produced estimates for six divisions of the country: Barisal, Chittagong, Dhaka, Khulna, Rajshahi, and Sylhet. Data were collected during 1 January–24 May 2004, and the study was conducted under the authority of the National Institute for Population Research and Training of the Ministry of Health and Family Welfare, Government of Bangladesh.

Households were selected by multistage sampling. The primary sampling units for the BDHS 2004 were subdivisions, known as enumeration areas, and the BDHS 2004 sample was a stratified multistage cluster sample consisting of 361 primary sampling units—122 in the urban area and 239 in the rural area (8). The BDHS 2004 used four sets of questionnaire that collected household and demographic information, including whether children aged less than five years (under-five children) in the household had received a vitamin A capsule within the last six months, receipt of tetanus-diphtheria-pertussis (DPT), oral poliovirus (OPV), and measles vaccines, and any history of neonatal, infant, or under-five child mortality in the family. For each child, mothers were asked if they had vaccination card for the child, and if so, to show the card to the interviewer. When the card was available, the interviewer entered the dates of immunization into the form in the questionnaire. If the card was not available or vaccinations were not recorded, mothers were asked questions to recall whether the child had received each vaccine. Twelve interviewing teams conducted fieldwork—each team consisting of one male supervisor, one female field editor, five female interviewers, two male interviewers, and one member of the logistics staff. Four quality-control teams of two persons each were used for monitoring the field teams (8).

The study protocol complied with the principles enunciated in the Helsinki Declaration (9). The field teams were instructed to explain the purpose of the survey to the mother or father, and data collection proceeded only after obtaining informed consent. Participation was voluntary, no remuneration was provided to subjects, and all subjects were free to withdraw at any stage of the interview. Data from the BDHS 2004 is in the public domain and was obtained through Measure DHS (10). The Institutional Review Board of the Johns Hopkins University School of Medicine approved the plan for secondary analysis of data.

In Bangladesh, all children aged 12 months and older (i.e. 12–59 months) are eligible to receive vitamin A supplementation every six months. Children of this age can receive vitamin A supplementation either through national immunization days or through the National Vitamin A Plus Campaign. Distribution of vitamin A capsules takes place twice a year through the national immunization days or National Vitamin A Plus Campaign, with specific dates announced in advance for fixed-site distribution. National vitamin A supplementation took place in October 2003 and February 2004.

The sample for analysis was restricted to households that had at least one child aged 18–59 months. The national vitamin A programme distributes capsules to children who are aged 12 months and older, and the question in the survey asked whether the child had received a capsule within the last six months. The youngest child, aged 18–59 months, in the household was used as the index of a child receiving a vitamin A capsule (i.e. households were not counted more than once because of clustering within households and children aged 18 months and older had all been eligible to receive a vitamin A capsule in the previous 6 months). The child growth standards of the World Health Organization were used as the reference growth curves (11). Stunting, underweight, and wasting were defined as height-for-age, weight-for-age, and weight-for-height z-scores <-2 respectively. Severe stunting, severe underweight, and severe wasting were defined as height-for-age, weight-for-age, and weight-for-height z-scores <-3 respectively. Maternal age was divided into quartiles. Maternal and paternal education was categorized as 0, 1–6, 7–9, and ≥10 years. The number of children in the family was used as an indicator of crowding. Multivariate logistic regression models were used for examining the relationship between maternal education and other variables and receipt of a vitamin A capsule in the last six months. Variables were included in the multivariate models if significant in univariate analyses. The p value of 0.05 was considered significant. Analyses of data were conducted using the SAS survey software (version 9.11) (SAS Institute, Cary, NC, USA).

## RESULTS

Of 3,745 children aged 18–59 months, 3,237 (86.4%) received a vitamin A capsule each within the last six months. The number and percentage of children who did not receive a vitamin A capsule in the last six months by demographic and other characteristics and the corresponding prevalence ratios (PRs) are shown in Table 1. The PR for receipt of a vitamin A capsule in the last six months was not significantly different from 1.00 for age and of the child's sex, or for underweight, severely-underweight, wasted, or severely-wasted children. The prevalence ratio for receipt of vitamin A in the last six months was significantly <1.00 for stunting, severe stunting, history of diarrhoea in the last two weeks in the children, and a history of infant mortality or under-five mortality in the child's family. The PR for receipt of vitamin A in the last six months was significantly >1.00 among children with older mothers, mothers or fathers with greater levels of education, and for those who received two or more doses of DPT, two or more doses of OPV, or measles immunization, and for children who came from families which owned a radio or a television.

**Table 1.** Characteristics of children and families by vitamin A capsule receipt in the last six months in Bangladesh

Characteristics*	Did not receive vitamin A (%)	Prevalence ratio (95% CI)
Age (months) of child		
18–23	66/421 (13.6)	1.00
24–35	173/1,044 (14.2)	0.99 (0.95–1.03)
36–47	139/999 (12.2)	1.02 (0.97–1.06)
48–59	130/773 (14.4)	0.99 (0.95–1.03)
Sex of child		
Female	254/1,581 (13.8)	1.00
Male	254/1,656 (13.3)	1.01 (0.98–1.03)
Maternal age (years)		
<20	70/375 (15.7)	1.00
20–22	109/653 (14.3)	1.02 (0.97–1.07)
23–26	104/805 (11.4)	1.05 (1.00–1.10)
27–30	90/587 (13.3)	1.03 (0.98–1.08)
≥31	132/787 (14.4)	1.02 (0.97–1.07)
Maternal education (years)		
0	246/1,228 (16.7)	1.00
1–6	181/1,204 (13.1)	1.04 (1.01–1.08)
7–9	57/502 (10.2)	1.07 (1.04–1.12)
≥10	21/270 (7.2)	1.11 (1.07–1.16)
Paternal education (years)		
0	216/1,136 (16.0)	1.00
1–6	136/849 (13.8)	1.03 (0.99–1.06)
7–9	40/364 (9.9)	1.07 (1.03–1.12)
≥10	29/359 (7.5)	1.10 (1.06–1.14)
Number of children in household		
1	57/469 (10.8)	1.00
2	118/760 (13.4)	0.97 (0.93–1.01)
3	101/665 (13.2)	0.97 (0.93–1.01)
≥4	232/1,343(14.7)	0.95 (0.92–0.99)
Stunting		
Yes	289/1,730 (14.3)	0.97 (0.95–1.00)
No	180/1,323 (12.0)	1.00
Severe stunting		
Yes	146/796 (15.5)	0.97 (0.94–1.00)
No	323/2,257(12.5)	1.00
Underweight		
Yes	232/1,400 (14.2)	0.98 (0.96–1.01)
No	249/1,714 (12.7)	1.00
Underweight		
Yes	232/1,400 (14.2)	0.98 (0.96–1.01)
No	249/1,714 (12.7)	1.00
Severe underweight		
Yes	84/508 (14.2)	0.99 (0.95–1.02)
No	397/2,606 (13.2)	1.00
Wasting		
Yes	67/390 (14.7)	0.98 (0.94–1.02)
No	390/2,589 (13.1)	1.00
Severe wasting		
Yes	12/104 (10.3)	1.04 (0.97–1.10)
No	445/2,875(13.4)	1.00
Diarrhoea in the last 2 weeks		
Yes	49/207 (19.1)	0.93 (0.88–0.99)
No	456/3,018 (13.1)	1.00
History of neonatal mortality		
Yes	51/257 (16.6)	0.96 (0.91–1.01)
No	457/2,980 (13.3)	1.00
History of infant mortality		
Yes	69/344 (16.7)	0.96 (0.92–1.00)
No	439/2,893 (13.2)	1.00
History of under-five child mortality in the family		
Yes	87/422 (17.1)	0.95 (0.91–0.99)
No	421/2,815 (13.0)	1.00
Diphtheria-pertussis-tetanus immunization		
No receipt	80/155 (34.0)	1.00
One dose	45/105 (30.0)	1.06 (0.92–1.22)
Two doses	40/164 (19.6)	1.23 (1.09–1.37)
Three doses	343/2,813 (10.9)	1.35 (1.23–1.48)
Oral poliovirus immunization		
No receipt	35/51 (40.7)	1.00
One dose	75/185 (28.9)	1.20 (0.99–1.45)
Two doses	39/119 (24.7)	1.27 (1.04–1.55)
Three doses	359/2,882 (11.1)	1.50 (1.26–1.79)
Measles immunization		
No	172/502 (25.5)	1.00
Yes	334/2,729 (10.9)	1.20 (1.14–1.25)
Ownership of radio/television		
No	336/1,856 (15.3)	1.00
Yes	172/1,381 (11.1)	1.05 (1.02–1.08)
Division		
Barisal	86/290 (22.8)	1.00
Chittagong	83/681 (10.9)	1.16 (1.09–1.23)
Dhaka	94/772 (10.9)	1.16 (1.09–1.23)
Khulna	57/453 (11.2)	1.15 (1.08–1.23)
Rajshahi	93/688 (11.9)	1.14 (1.07–1.21)
Sylhet	95/353 (21.2)	1.02 (0.95–1.10)

*There were missing data (n) for the following variables: maternal age (33), maternal education (36), paternal education (616), stunting (223), underweight (150), wasting (309), diarrhoea (15), oral polio immunization (102), and measles immunization (8);

CI=Confidence interval

Higher level of maternal education, greater maternal age, and ownership of a radio or a television were significantly associated with the child receiving a vitamin A capsule in the last six months in a multivariate model, adjusting for location (Table 2). When under-five child mortality was added to the previous multivariate model, the relationship between non-receipt of a vitamin A capsule and previous under-five child mortality in the family was of borderline significance (PR 1.03, 95% CI 0.99–1.08).

**Table 2.** Multivariate logistic regression analysis of factors associated with child's receipt of a vitamin A capsule in the last six months in Bangladesh*

Characteristics	Prevalence ratio (95% CI)
Maternal age (years)	
<20	1.00
20–22	1.02 (0.76–1.54)
23–26	1.05 (1.01–1.10)
27–30	1.05 (1.00–1.10)
≥31	1.04 (1.00–1.10)
Maternal education (years)	
0	1.00
1–6	1.05 (1.02–1.08)
7–9	1.08 (1.04–1.12)
≥10	1.09 (1.04–1.13)
Ownership of radio or television	1.03 (1.00–1.05)

*Model adjusted for variables above and for division;

CI=Confidence interval

## DISCUSSION

Data from the BDHS 2004 showed that the coverage of the vitamin A supplementation programme to prevent morbidity, mortality, and blindness in preschool children was relatively high in Bangladesh, exceeding the 85% coverage rate recommended by the World Bank (12). The findings of the present study showed that the remaining 14% of preschool children who missed a vitamin A capsule in the last six months were more likely to come from families that had higher under-five child mortality. When under-five child mortality was included in a multivariate model adjusting for other covariates, the relationship between non-receipt of a capsule and previous under-five child mortality in the family was of borderline significance. Children missed by the programme may be those who could probably benefit the most, given the higher history of under-five child mortality in their families.

In the present study, children who were missed by the vitamin A capsule-distribution programme were less likely to have received DPT, OPV, and measles immunization. In the national vitamin A programme in Indonesia, there was also a similar, lower coverage among children who missed childhood immunizations (13). The lack of immunization places children who missed vitamin A at an even higher risk of morbidity and mortality from vaccine-preventable infectious diseases. Vitamin A deficiency increases the risk of morbidity due to measles, including the severity of diarrhoeal disease, measles-related pneumonia, blindness, and mortality (14). These findings suggest that the demographic factors that impact a child's participation in vitamin A supplementation programmes may also impact participation in other public-health interventions.

A previous report from Helen-Keller International showed that the coverage of the vitamin A supplementation programme was below 70% in the Chittagong Hill Tracts, a district within Chittagong division (15). In the present study, the overall coverage in Chittagong division was 89.1%, and separate figures were not available for the Chittagong Hill Tracts alone.

Results of a previous study in Indonesia showed that children who were missed by the vitamin A programme were more likely to be stunted, underweight, or wasted (13). In the present study in Bangladesh, there were significant differences in the prevalence of stunting and severe stunting between children who did and did not receive a vitamin A capsule in the last six months. Stunted children are more likely to suffer from vitamin A deficiency disorders and have higher morbidity due to infectious diseases (4), and they represent a vulnerable group which could benefit if the coverage of the vitamin A programme could be expanded.

The national vitamin A programme was initially established by the Bangladesh Programme for the Prevention of Blindness with support from the United Nations Children's Fund in 1973 (16). Supplementation of vitamin A has been shown to protect against clinical vitamin A deficiency, as indicated by nightblindness, among preschool children in Bangladesh (16). In Indonesia and Viet Nam, the expanded coverage of the national vitamin A supplementation programme was also accompanied with large reductions in hospital admissions for xerophthalmia (4, 17). The national vitamin A supplementation programme in Ethiopia achieved a coverage of less than 50% in 2005, and children who did not receive vitamin A in the last six months were more likely to come from families with lower maternal and paternal education (18).

The findings of the present study suggest that maternal education is an important factor relating to receipt of a vitamin A capsule. A higher level of formal education achieved by girls may be a key factor in breaking the intergenerational cycle of malnutrition and poverty (19). Since younger maternal age was also associated with the lower coverage, further efforts are, thus, required by the vitamin A supplementation programmes to reach young, uneducated primigravida mothers. Also, children of households of higher socioeconomic status were more likely to have received a vitamin A capsule. The coverage ranged from 77.2% in Barisal and 78.8% in Sylhet to 89.1% in Dhaka and Chittagong divisions, and there were variations between areas within each division. Thus, it is also important for programmes to identify areas with a lower coverage and implement measures to increase the coverage and, in particular, ensure the coverage of the most vulnerable children (stunted, of lower socioeconomic background, and with uneducated and younger mothers).

Worldwide, 9.7 million children die each year; most deaths occur from preventable causes, and nearly all deaths occur in poor countries (20). About 63% of deaths of children could be prevented with full implementation of the few known and effective interventions to reduce child mortality, such as vitamin A supplementation (5). The reduction of under-five child mortality by two-thirds between 1990 and 2015 is one of the MDGs that was endorsed by 189 countries in September 2000 (5). Although the coverage of the vitamin A capsule programme in Bangladesh is relatively high, efforts to reach 14% of the children missed by the programme may yield substantial benefit in reducing child deaths and nutritional blindness.

## ACKNOWLEDGEMENTS

The study was supported by the Eye Foundation of America and a Lew R. Wasserman Merit Award from Research to Prevent Blindness to Dr. Semba.
